# Characterization of AKT independent effects of the synthetic AKT inhibitors SH-5 and SH-6 using an integrated approach combining transcriptomic profiling and signaling pathway perturbations

**DOI:** 10.1186/1471-2407-10-287

**Published:** 2010-06-14

**Authors:** Till Krech, Margarethe Thiede, Ellen Hilgenberg, Reinhold Schäfer, Karsten Jürchott

**Affiliations:** 1 Laboratory of Molecular Tumor Pathology, Institute of Pathology, Charité, Universitätsmedizin Berlin, Charitéplatz 1, D-10117 Berlin, Germany; 2 Institute of Pathology, Hannover Medical School, Carl-Neuberg-Str. 1, D-30625 Hannover, Germany; 3 Institute for Theoretical Biology, Humboldt-University, Invalidenstrasse 43, D-10115 Berlin, Germany

## Abstract

**Background:**

Signal transduction processes mediated by phosphatidyl inositol phosphates affect a broad range of cellular processes such as cell cycle progression, migration and cell survival. The protein kinase AKT is one of the major effectors in this signaling network. Chronic AKT activation contributes to oncogenic transformation and tumor development. Therefore, analogs of phosphatidyl inositol phosphates (PIAs) were designed as new small drugs to block AKT activity for cancer treatment. Here we characterize the biological effects of the PIAs SH-5 and SH-6 in colorectal cancer cell lines.

**Methods:**

Serum-starved or serum-supplemented human colorectal cancer cell lines SW480, HT29 and HCT116 were exposed to SH-5 and SH-6. AKT activation was determined by western blotting. Cell viability was assessed using a colorimetric XTT-based assay, apoptosis and cell cycle changes were monitored by FACS analysis. The dynamics of cell morphology alterations was evaluated by confocal and time-lapse microscopy. Transcriptional changes due to inhibitor treatment were analyzed using Affymetrix HG-U133A microarrays and RT-PCR.

**Results:**

While the PIAs clearly reduce AKT phosphorylation in serum starved cells, we did not observe a significant reduction under serum supplemented conditions, giving us the opportunity to analyze AKT independent effects of these compounds. Both inhibitors induce broadly the same morphological alterations, in particular changes in cell shape and formation of intracellular vesicles. Moreover, we observed the induction of binucleated cells specifically in the SW480 cell line. Gene expression analysis revealed transcriptional alterations, which are mostly cell line specific. In accordance to the phenotype we found a gene group associated with mitosis and spindle organization down regulated in SW480 cells, but not in the other cell lines. A bioinformatics analysis using the Connectivity Map linked the gene expression pattern of the inhibitor treated SW480 cells to PKC signaling. Using confocal laser scanning microscopy and time lapse recording we identified a specific defect in the last step of the cytokinesis as responsible for the binucleation.

**Conclusions:**

The PIAs SH-5 and SH-6 impinge on additional cellular targets apart from AKT in colorectal cancer cells. The effects are mostly cell line specific and have an influence at the outcome of the treatment. In view of potential clinical trials it will be necessary to take these diverse effects into consideration to optimize patient treatment.

## Background

A wide variety of physiological processes is controlled by sequestering regulatory proteins to specific membrane domains. Derivates of phosphatidyl inositol play a crucial role in this process. The inositol ring can be phosphorylated at the 3^rd^, 4^th ^or 5^th ^position, resulting in different phosphatidyl inositol phosphates. During the last decades the signal transduction processes mediated by the diverse phosphatidyl inositol phosphates have been deciphered. Phosphatidyl inositol(4,5)-bisphosphate (PI(4,5)P_2_) is synthesized by type I (PIPKI) or type II (PIPKII) kinases using either phosphatidyl (4)-phosphate or phosphatidyl (5)-phosphate as a substrate [1]. PI(4,5)P_2 _is an adaptor for several proteins containing a PDZ domain, e.g. phospholipase C (PLC), syntenin and the tight junction protein 1 (ZO-1), and is involved in the regulation of the cytoskeleton [2], cytokinesis [3] and in the stabilization and activation of integral membrane proteins such as transporters and ion channels. Furthermore, PI(4,5)P_2 _can be either hydrolyzed to the secondary messengers diacylglycerol (DAG) and inositol (1,4,5)-trisphosphate (IP_3_), or further phosphorylated by PI3 kinases to phosphatidyl inositol (3,4,5)-trisphosphate (PI(3,4,5)P3), an important activator of the AKT signaling pathway [4].

A great body of evidence suggests that the oncogenic activation of AKT contributes to cellular transformation and influences tumor development and progression [5-7]. Therefore, AKT is an interesting and promising target for pharmacological intervention [8]. Several synthetic AKT inhibitors like perifosine, GSK2110183, and RX-0201 entered phase I and II clinical trials. During the last years, synthetic analogs of phosphatidyl inositol phosphates (PIAs) were developed to block AKT activity in tumor cells [9].

In our study, we used two synthetic phosphatidyl inositol phosphate analogs (SH-5 and SH-6), which lack the hydroxyl group at position three of the inositol ring and display modified aliphatic side chains conferring a higher metabolic stability [9]. Previous cell culture studies have suggested that the two compounds prevent AKT activation by interfering with its phosphatidyl inositol binding domain and thereby induce apoptosis [10]. Most of the experiments were done either under moderate serum conditions (5%) or after serum starvation (0.1%) [11,12]. To mimic the conditions in tumors exhibiting a high angiogenic activity, resulting in a growth factor-rich micro-milieu, we decided to test the effects of PIAs under standard conditions in the presence of 10% fetal calf serum. We verified the inhibition of AKT in three colorectal cancer cell lines deprived of growth factors, but did not observe a reduction of AKT activity under normal cell culture conditions including fetal calf serum at standard concentration. Despite the missing effects on AKT activity under full supplemented cell culture conditions, we detected a broad range of morphological and transcriptional alterations, indicating that these compounds affect other sub cellular targets too. Most remarkably, both compounds mediated a defect in the abscission, the last step of cytokinesis, in the SW480 cells, resulting in binucleation.

## Results

### The phosphatidyl inositol phosphate analogs SH-5 and SH-6 induce morphological alterations in colorectal cancer cells

To study the biological effects of phosphatidyl inositol phosphate analogs (PIA) on phosphoinositide dependent signaling we chose three well established colorectal cancer cell lines as a model. First, because a large fraction of colorectal cancer specimens and cell lines display mutations of the PIK3CA gene (which codes for p110alpha, a catalytic subunit of PI3K) and second, because colorectal cancer specimens show increased PIP3 levels compared to control tissues, both suggesting a pivotal role for phosphoinositide signaling in colorectal cancer [13,14].

**Figure 1:**
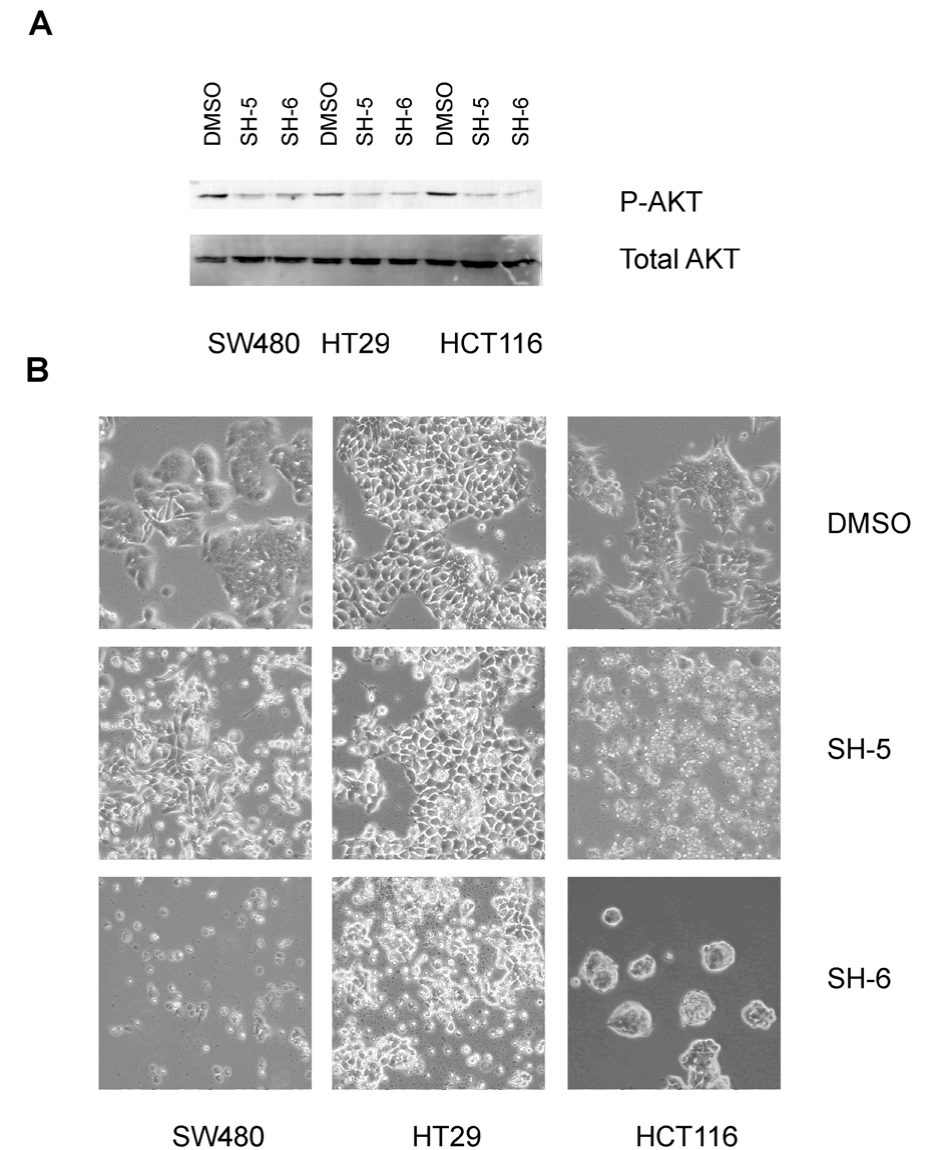
**PIAs inhibit AKT phosphorylation after serum starvation**. (A) Western blot analysis of cells serum starved for 24 hours prior to incubation for two hours with SH-5 and SH-6, respectively. The phosphorylation status of AKT was determined using specific antibodies. The detection of total AKT served as a loading control. (B) Morphological alterations induced in serum starved cells after 24 hours of treatment with SH-5 and SH-6, respectively. DMSO was used as a solvent control.

SW480, HT29 and HCT116 cells harbor different kinds of oncogenic mutations which reflect the common spectrum of alterations in colorectal cancers [15]. The cells were serum starved for 24 hours, followed by treatment with either DMSO or one of the phosphatidyl inositol phosphate analogs (SH-5, SH-6) for two hours. We observed a reduction of AKT phosphorylation in all of the three cell lines, according to the proposed function of the PIAs as AKT inhibitors (Figure 1A). A further incubation of the cells for 24 hours resulted in rounding up of the cells and induction of cell death (Figure 1B).

**Figure 2:**
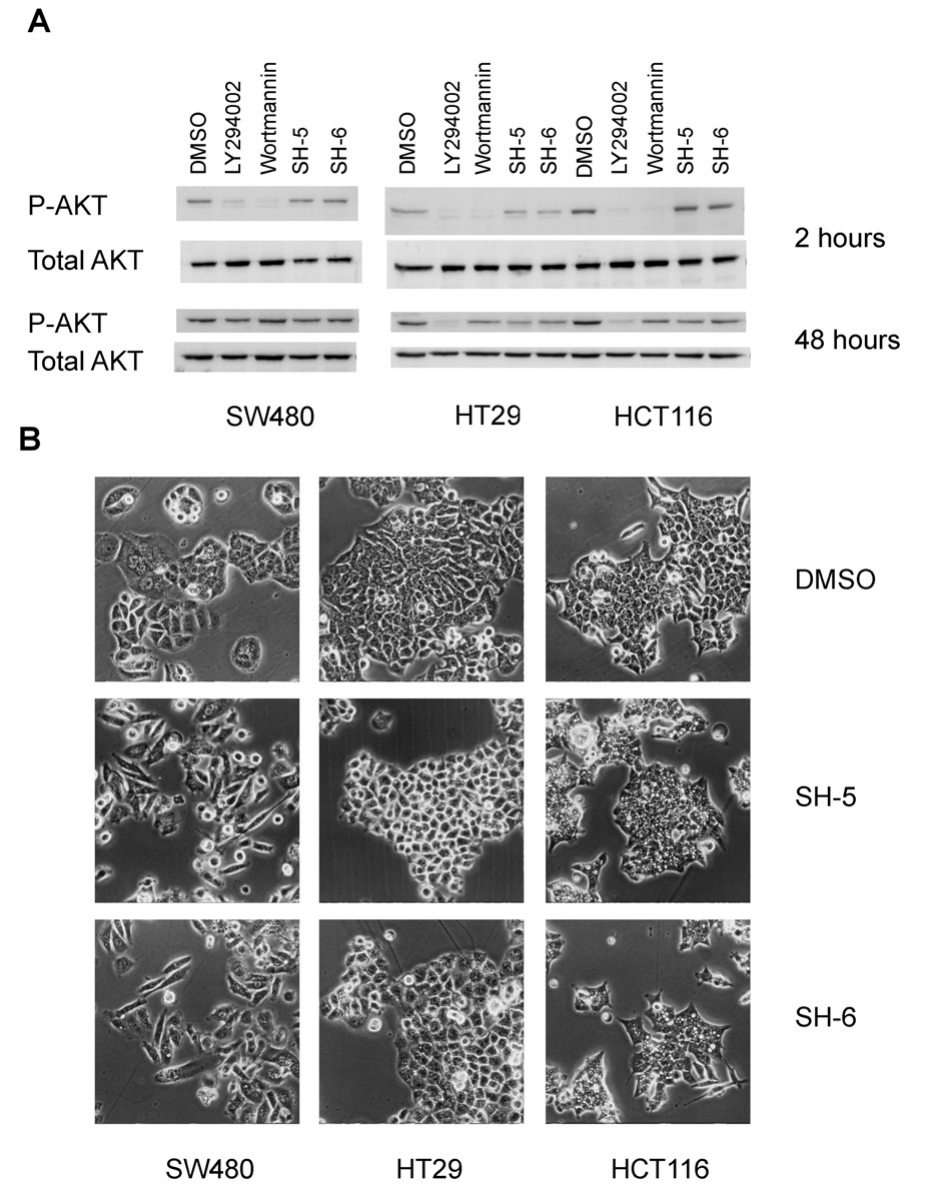
**PIAs did not reduce AKT activity under full supplemented culture conditions**. (A) Western blot analysis of colorectal cancer cells treated with either specific PI3 kinase inhibitors (LY294002, Wortmannin) or PIAs (SH-5, SH-6) for 2 and 48 hours, respectively. DMSO served as the solvent control. Levels of phosphorylated AKT were determined using specific antibodies. Total AKT was determined as a loading control. (B) Morphological alterations induced by the PIAs after 48 hours of treatment.

In contrast, we did not observe any significant effect on the phosphorylation status of AKT under cell culture conditions including 10% fetal calf serum. Using two well characterized PI3 kinase inhibitors as positive control, we observed a strong reduction of AKT phosphorylation after two hours of incubation under the same conditions. Whereas wortmannin appeared to act transiently due to rapid decay/inactivation, the effect of a single treatment with LY294002 lasted for at least 48 hours in two of these cell lines (Figure 2A).

Despite the lack of any clear effect of the PIAs on AKT phosphorylation under normal serum conditions, we observed clear morphological alterations of the treated cells. In SW480 cells, SH-5 and SH-6 caused a spindle-like morphology and increased cell scattering. The formation of large cytoplasmic vesicles was prominent in the HT29 and HCT116 cells (Figure 2B). For fully supplemented media conditions these findings suggest additional targets of the PIAs apart from AKT.

### A genome-wide identification of transcriptional targets associated with SH-5 and SH-6 treatment

**Figure 3:**
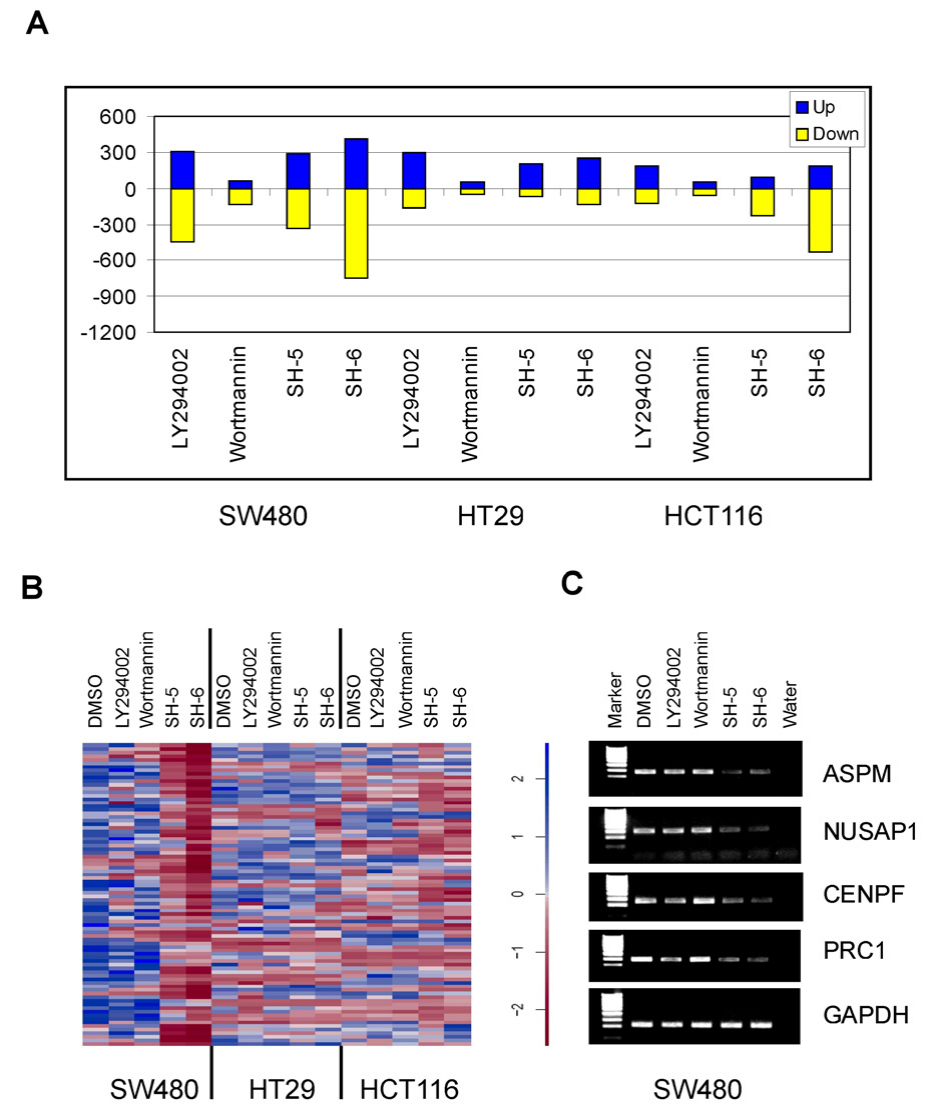
**Distinct alterations of gene expression caused by the PIAs**. (A) Graphical display of up- and down-regulated genes (fold change > = 2) after 48 hours of treatment with either the PI3kinase inhibitors or the PIAs. (B) Heatmap of probe sets which are associated with the mitotic cell cycle (GO-annotation GO:0000278) and down regulated in the SW480 cells by SH-5 and SH-6, respectively. (C) Verification of down regulated genes in SW480 cells using RT-PCR.

Our observations raised the question, which other targets might be affected by the PIAs. Such targets might contribute to anti-cancer treatment or unwanted side effects. In order to identify additional targets of the PIAs, we performed a genome-wide expression analysis of control cells and cells treated with the PI3-Kinase inhibitors or PIAs for 48 hours. RNA was extracted as described in methods and used to interrogate HG-U133A microarrays. We determined probesets of differentially expressed genes (fold change > = 2) in comparison to the DMSO control (Figure 3A). We identified a distinct set of target genes of the PIAs specific for each cell line. In addition, there is a partial overlap of genes down-regulated by SH-6 between the SW480 and the HCT116 cells. Most of the transcriptional alterations induced by the phosphatidyl inositol analogs were found in the SW480 cells. We observed only a limited number of transcriptional changes in each cell line treated with wortmanin, consistent with the observation, that wortmanin will be inactivated within 48 hours. In contrast, we found a higher number of differentially expressed genes after LY294002 treatment. The number of up-regulated genes compared to the down-regulated genes is higher in HCT116 and HT29 cells. Although AKT is active again in SW480 cells after 48 hours of treatment with LY294002, the overall number of regulated genes is higher than in the other two cell lines. These transcriptional changes suggest a persistent action of LY-294002 on SW480 cells, reshaping the signaling network and thus finally leading to the reconstitution of AKT-activity.

We performed an *in silico *analysis of the annotated biological features of differentially expressed genes (GO-annotation) using Expander 4.0 [16] in order to find out overrepresented functional groups of genes affected by the PIAs (Additional file 1). A coordinated down-regulation of genes associated with the mitotic cell cycle, especially M-phase, was peculiar to the SW480 cells treated with SH-5 or SH-6 (Figure 3B). We verified the down regulation of four genes out of this group with RT-PCR (Figure 3C). Moreover, we found that genes linked to the translational machinery and to cell migration were up-regulated in the SW480 cells. The PIAs caused the up-regulation of genes encoding components of the sterol, isoprenoid and cholesterol metabolic process in HCT116 cells. Furthermore, we identified an overrepresentation of genes involved in the immune response against viruses among the up-regulated genes in the HT29 cells. In contrast to that, the number of over represented GO-terms in the expression profiles of wortmanin or LY294002 treated cells was quite small (Additional file 1).

### PIAs induce binucleation in SW480 cells

The treatment of the SW480 cells with PIAs resulted in a down-regulation of a set of genes involved in the progression of the M-phase of the cell cycle and the organization of the mitotic spindle. Therefore, we expected defects in the progression of SW480 cells through this cell cycle phase. We determined the proliferation rate of cells after the SH-5 or SH-6 treatment using a colorimetric XTT-assay. We observed only a small decrease in cell proliferation indicating that the down-regulation of target genes affecting mitosis was insufficient to induce a cell cycle block (Additional file 2A).

Accordingly, we did not obtain any evidence for the induction of apoptosis by using FACS analysis (Additional file 2B).

**Figure 4:**
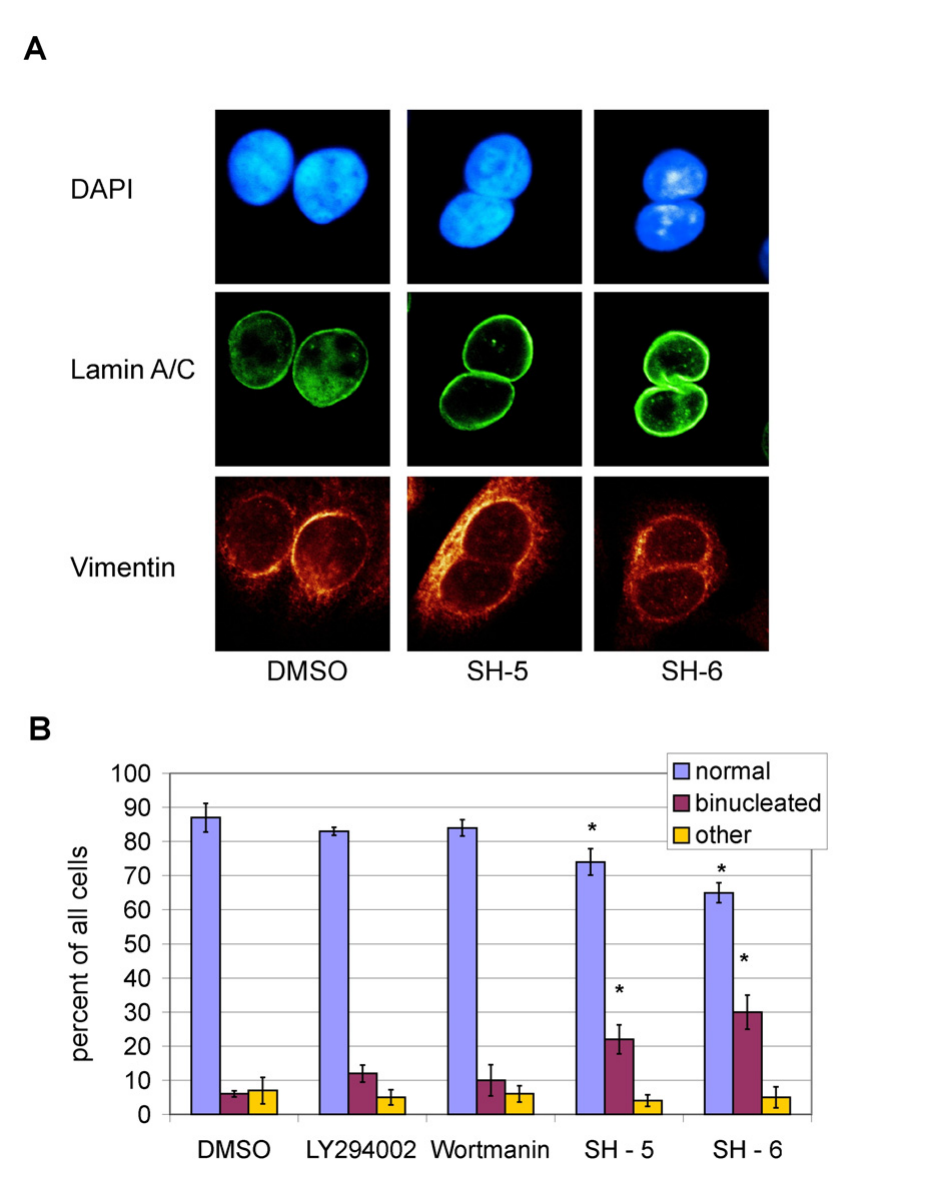
**PIAs induce binucleation in SW480 cells**. (A) Confocal Laser scanning images of cells treated with SH-5, SH-6 or DMSO. The green staining indicates Lamin A/C, the red Vimentin and the blue DAPI. (B) Percentage of binucleated cells determined microscopically after treatment with the indicated inhibitors or the DMSO control. The total number of cells was set to 100. Data sets were analyzed using the Student's t-Test. A p-value < 0.05 was considered as significant and marked by asterisks.

**Figure 5:**
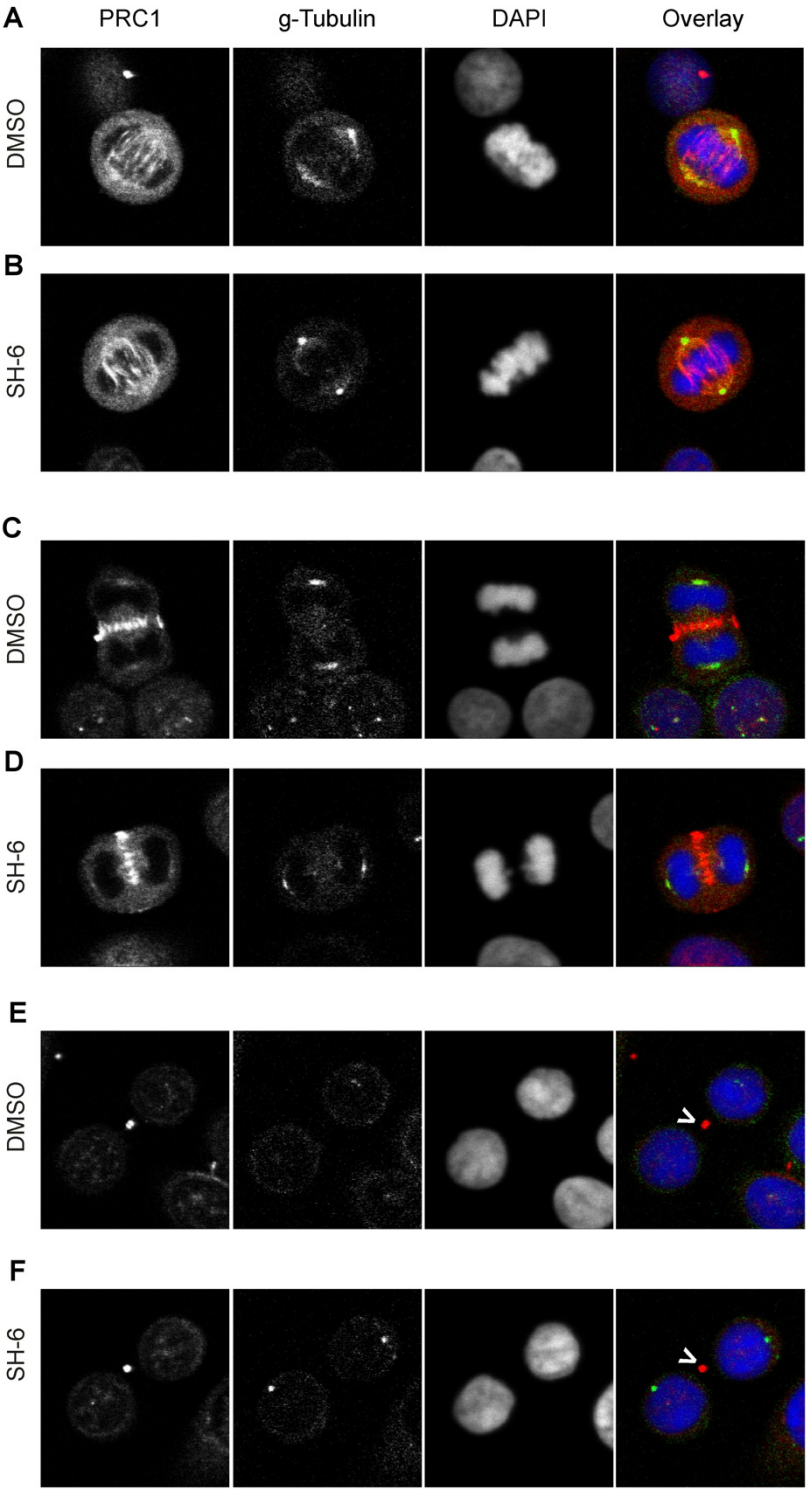
**Absence of visible mitotic defects in PIA-treated SW480 cells**. Fixed SW480 cells preincubated with either DMSO (A, C and E) or SH-6 (B, D and F) for 48 hours were stained with anti-PRC1 (red), anti-γ-Tubulin (green) and DAPI (blue). We selected typical cells in different stages of the mitosis based on the DAPI staining: (A) and (B) cells in metaphase, (C) and (D) cells in anaphase, (E) and (F) cells in telophase (white arrows mark the midbodies).

Next we analyzed pretreated SW480 cells using confocal laser scanning microscopy to reveal alterations induced by the PIAs. We discovered a marked increase of binucleated cells after treatment with SH-5 or SH-6, compared to the vehicle-treated control population (Figure 4A, B). To characterize the mechanism underlying this increase of binucleated cells we investigated the different steps of the mitotic division. Cells were stained with antibodies directed against γ-Tubulin, which is an integral part of the centrosomes and with antibodies against protein regulator of cytokinesis 1 (PRC1). PRC1 colocalizes with the mitotic spindle during metaphase (Figure 5A, B) and relocalizes to the cleavage furrow in anaphase (Figure 5C, D). In the succeeding telophase, PRC1 is part of the midbody between the emerging daughter cells (Figure 5E, F). We did not detect any significant difference between treated and control cells through-out these cell cycle phases, suggesting that the defects must occur to a final stage of cell division. In addition, we did not observe an increasing number of chromosome bridges which might explain the failure of nuclear division.

**Figure 6:**
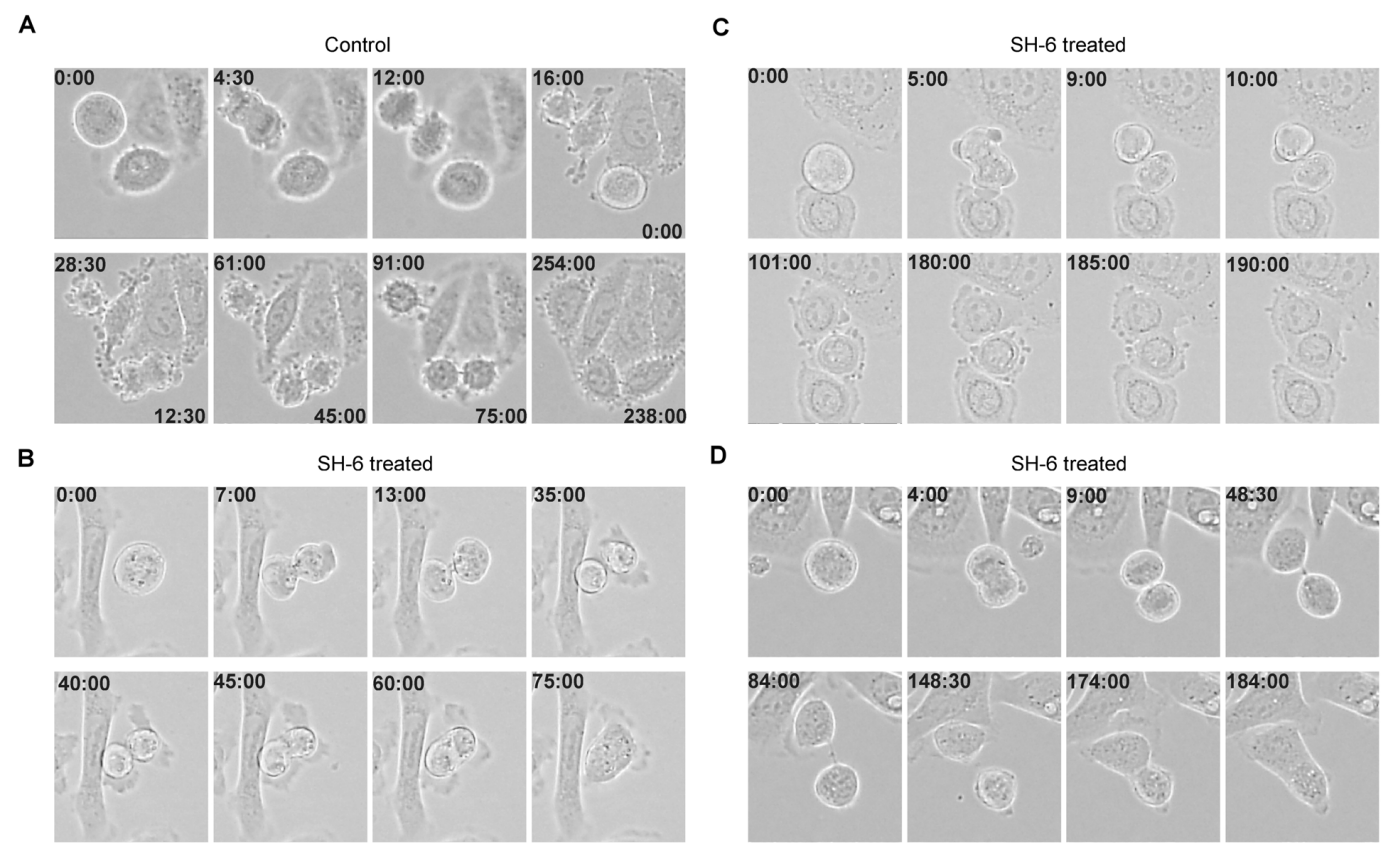
**Time lapse recording revealed failures in abscission of PIA-treated SW480 cells**. Time lapse recording was started 24 hours after the incubation of SW480 cells with either SH-6 or DMSO as a vehicle control. Images were made every 30 seconds and aligned to movies. The bright field images token out of the time lapse movies show dividing SW480 cells in the time from 24 to 48 hours after treatment. Control (A) as well as SH-6 treated (B - D) cells progress similar through mitosis until cytokinesis. The daughter cells of the control samples will be separated during the cytokinesis, resulting in two individual cells (A). In contrast, the SH-6 treated cells fail to separate the bridge between the daughter cells, resulting in re-fusion and forming of binucleated cells (B - D). The time at the image corners is indicated in minutes.

To better define the exact time course of cell cycle distortion, we performed time-lapse analysis of treated and control cells. The cells regularly progressed through mitosis until reaching the last step of cytokinesis (Figure 6A, Additional file 3). During this step, called abscission, the bridge between the daughter cells is normally disrupted. PIA treated SW480 cells regularly performed nuclear division and formed daughter cells initially. However, in contrast to the control cells, the intercellular bridge remained stable for up to three hours with consecutive re-fusion, giving rise to binucleated cells (Figure 6B-D, Additional files 4, 5 and 6). In summary these findings demonstrate that the treatment with PIAs specifically interferes with abscission in SW480 cells.

### The PIA-mediated binucleation in SW480 cells is independent of a general PLC inhibition

**Figure 7:**
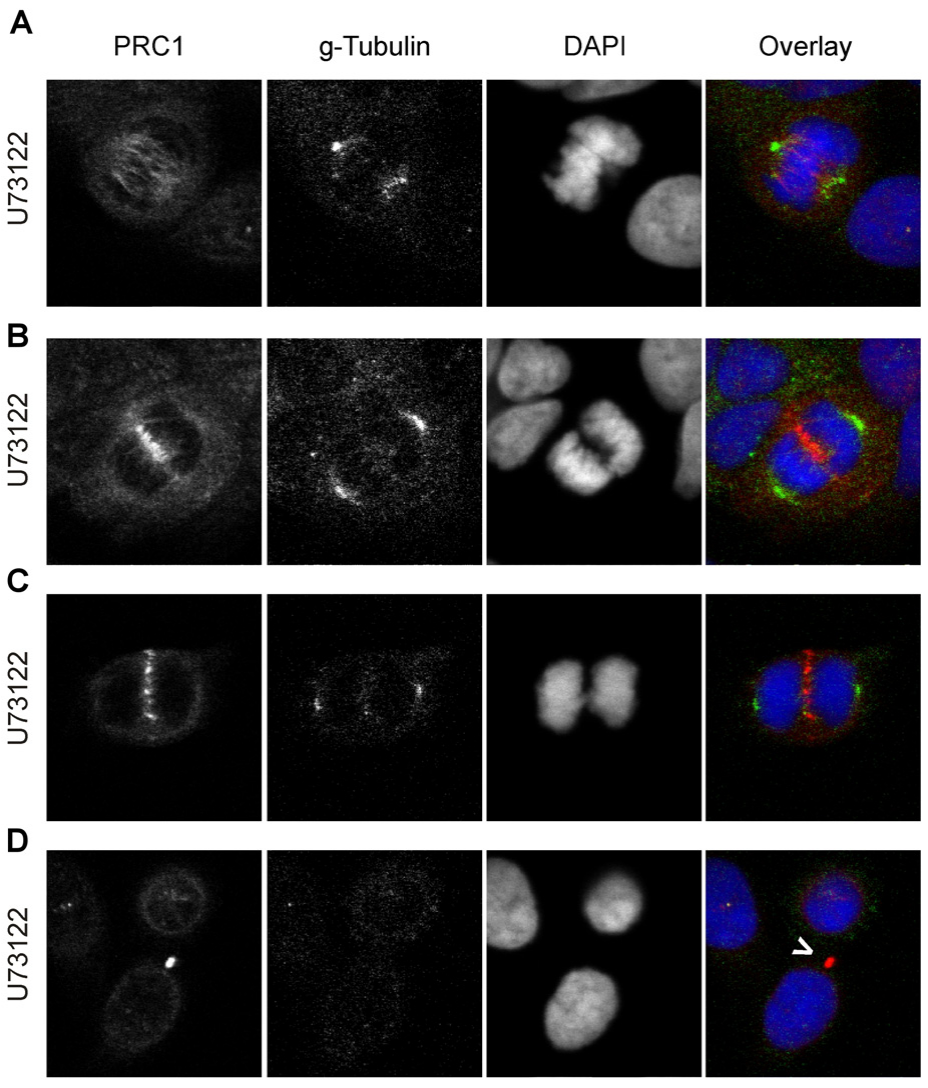
**Induction of mitotic defects by the specific PLC inhibitor U73122**. Fixed SW480 cells preincubated with the specific PLC inhibitor U73122 for 48 hours were stained with anti-PRC1 (red), anti-γ-Tubulin (green) and DAPI (blue). We selected typical cells in different stages of the mitosis based on the DAPI staining: (A) Cells in metaphase with defect metaphase plate, (B) Cells in anaphase with failure in chromosome segregation, (C) Cells in anaphase with chromosome bridge, (D) Cells in telophase, daughter cell nuclei have different size, indicating failure in chromosome segregation (the white arrow marks the midbody between the daughter cells).

Since AKT activity does not seem to be reduced significantly by PIAs under normal serum condition, we looked for other potential effector molecules. The phospholipase C (PLC) binds to PI(4,5)P_2 _and hydrolyzes it to DAG and IP_3_. PLC is localized at the cleavage furrow during cytokinesis and is involved in the regulation of this process [17]. Therefore we hypothesized that the metabolically stable PIAs might be able to bind to and inhibit PLC. We incubated SW480 cells with the PLC inhibitor U73122 for 48 hours and fixed the cells as described above. We analyzed the samples by confocal laser scanning microscopy after staining them with anti-PRC1, anti-γ-Tubulin antibodies and DAPI. We observed various defects during mitosis of SW480 cells treated with U73122. These including defects in forming the metaphase plate (Figure 7A), in chromosome segregation (Figure 7B) and an increase in the fraction of cells with chromosome bridges (Figure 7C). In addition to that, we detected differentially sized daughter cells indicating defects during karyogenesis (Figure 7D). However, in contrast to the PIAs, we did not found any evidence for the induction of binucleated cells after U73122 treatment. We conclude that the PIAs cause binucleation by a mechanism independent of global PLC activity.

### A Connectivity Map analysis suggests the PKC signaling pathway as a PIA target

**Figure 8:**
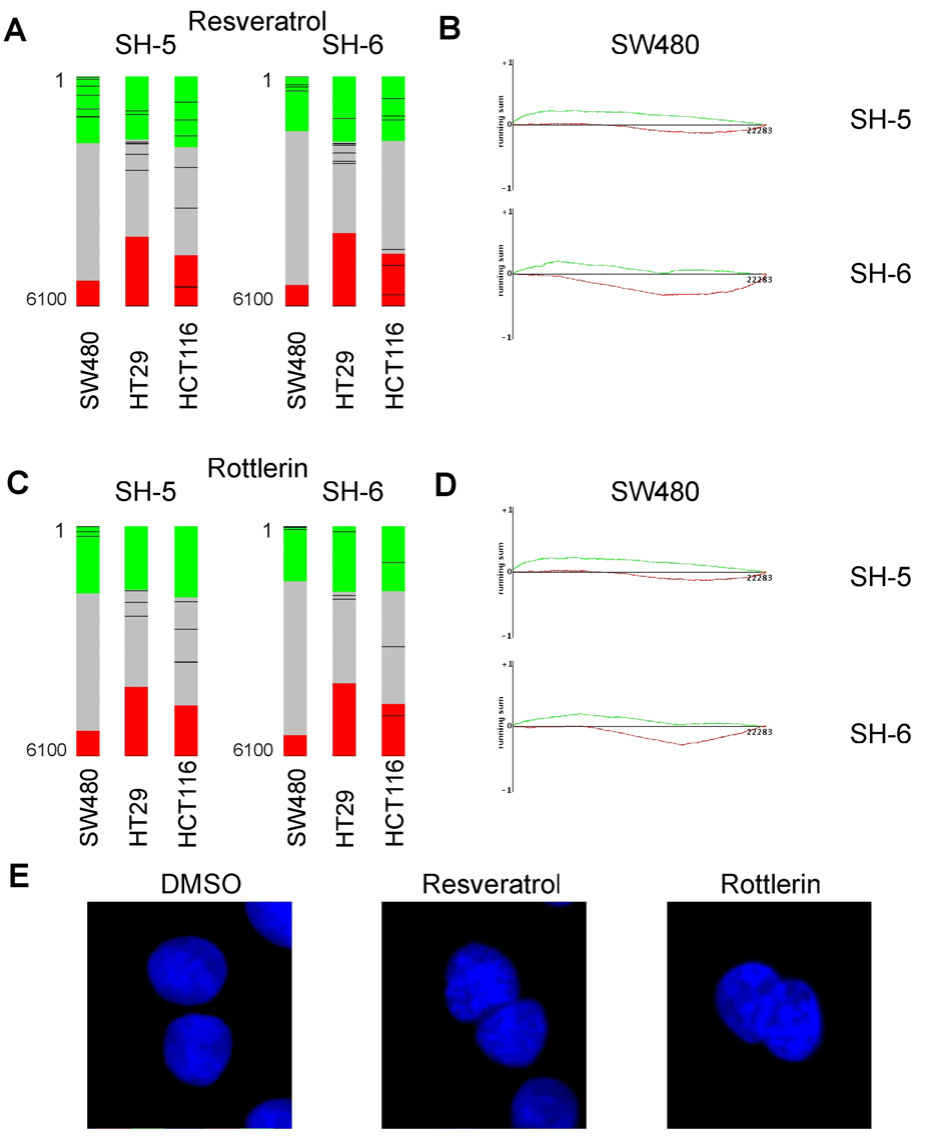
**Similarities of gene expression profiles in SW480 cells treated with PIAs and PKC signaling pathway inhibitors**. (A) and (C) Each of the barviews is composed of 6100 individual treatment instances from the Connectivity Map (build 02) database ranked according to their similarity to the SH-5 or SH-6 gene signatures. The green area illustrate a positive, the red a negative and the gray field no enrichment with the indicated signatures. Black lines corresponding to distinct individual instances of MCF7 cells treated with either Resveratrol (A) or Rottlerin (C). Please note, that all marked instances are among the top ranking in SW480 cells, whereas they are broadly distributed all over the barviews in the other cell lines. (B) and (D) The enrichment of PIA specific gene signatures is demonstrated for the best matched individual treatment instances. Gene probes which are induced (green) or repressed (red) by PIAs were ordered according to their differential expression in MCF7 cells caused by the treatment with Resveratrol and Rottlerin, respectively (x-axis). The running sum of the Kolomogorov-Smirnov test is shown at the y-axis. Most genes which are induced by the PIAs appear early in the treatment profile, whereas the converse is true for repressed genes. (E) The treatment of SW480 cells with either Resveratrol or Rottlerin for 48 hours results in binucleated cells as detected by DAPI staining.

In order to find out more about the molecular basis of binucleation in the SW480 cells, we took advantage of the Connectivity Map (build 02), a web implemented database of 6,100 gene expression profiles representing the treatment of different cells with 1,309 bioactive compounds of mostly known activity [18]. This database uses a Kolmogorov-Smirnov test statistic to rank order the 6,100 individual treatment instances according to their similarity to the user-provided signature of up- and down-regulated genes. A detailed summary of this analysis is shown in Additional files 7 and 8. Several of the top ranking instances related to PIA-treatment of SW480 cells corresponded to treatments with compounds known to interfere with the PIP2 (e.g. Resveratrol), the Ca^2+ ^(e.g. Thioridazine) or the PKC signaling (e.g. Rottlerin) (Figure 8A-D). Since PKC activity is depended on Diacylglycerol (DAG), a product of the PIP2 hydrolyses, and Ca^2+ ^levels, these similarities hint at PKC signaling pathway as a potential PIA target. Moreover, we found instances corresponding to treatments with antagonists of the dopamine receptor under the highest ranking candidates (e.g. Prochlorperazine). Dopamine receptors are G-protein coupled receptors which may also converge on the PKC signaling pathway [19]. In order to prove if the treatment with the suggested compounds results in a similar phenotype as with PIAs, we incubated SW480 cells for 48 hours with Resveratrol and Rottlerin, respectively. Microscopic analysis of treated cells revealed an increase of binucleation with both compounds (Figure 8E).

## Discussion

Genome-wide expression profiling of inhibitor-treated colorectal cancer cells revealed some unexpected and novel features of two synthetic AKT inhibitors. The most remarkable alteration was the down-regulation of genes associated with mitosis in the SW480 cell line, accompanied by the induction of binucleation. Using confocal laser scanning microscopy and time-lapse recordings, we identified a specific defect during the abscission of the daughter cells as the cause of binucleation. Perturbation studies with pharmacological inhibitors suggested an involvement of PKC signaling in this process.

Expression profiling of treated SW480 cells demonstrated down-regulation of genes associated with mitosis. The effect of this reduced gene expression on cell growth was surprisingly weak, indicating that the remaining expression of most of these genes was sufficient to allow cell cycle progression. In addition, the XTT proliferation assay is based on a metabolic process, in which the tetrazolium salt XTT is cleaved to form soluble colored formazan. It is well established that metabolic activity is highly correlated with the number of cells in the assay (Roche). Since PIA-treated SW480 cells divide until the last step of the abscission, they behave like two cells after re-fusion regarding the metabolic activity. We assume that binucleated cells retain this metabolic activity.

Despite the down-regulation of several genes associated with spindle formation and genes with critical functions during mitosis, we observed no defects in the mitosis until the last step of the abscission. The mitotic spindle is not only implicated in chromosome segregation during mitosis but also affects the crucial steps of cytokinesis. The central spindle complex concentrates key regulators of the cytokinetic machinery, thus providing the basis for the final step of cell division. As spindle assembly, chromosome segregation and cytokinesis require complex protein interactions and possibly critical thresholds of individual components, not necessarily reflected in mRNA levels, the deregulation of mitotic spindle genes might affect cytokinesis without affecting chromosomal segregation.

We validated the down-regulation of ASPM (abnormal spindle homolog, microcephaly associated), NUSAP1 (nucleolar and spindle associated protein 1), PRC1 (protein regulator of cytokinesis 1) and CENPF (centromere protein F (mitosin)) which are all necessary for proper mitotic cell division. The NUSAP1 protein is localized at the central spindle tubules during mitosis and gene silencing by RNA interference resulted in defects of chromosome segregation and cytokinesis [20,21]. ASPM is located at the spindle poles or centrosomes during mitosis [22]. Mutations in ASPM are associated with autosomal recessive microcephaly as a consequence of failures in the chromosome segregation. The knock-down of CENPF with specific siRNA caused defects in metaphase chromosome alignment, anaphase chromosome segregation and cytokinesis [23]. PRC1 encodes a microtubule bundling protein with an essential function in the formation of the contractile ring in the cleavage furrow and in cytokinesis. The knock-down of PRC1 results in the induction of binucleated cells as a result of defects during abscission [24,25]. In contrast to the decreased RNA expression, we detected comparable levels of PRC1 protein in immune fluorescence analysis of treated and control cells, suggesting an additional control at the level of translation or protein stability that might compensate for transcriptional down-regulation. Based on this observation we propose that PRC1 is not the major cause of binucleation in our cell model. Since expression profiling showed down-regulation of multiple mitosis-associated genes it is likely that the binucleation in SW480 cells is a result of a multi-gene effect rather than due to the perturbation of a single gene. The synthetic compounds SH-5 and SH-6 used in our study are thought to work as competitive inhibitors of the naturally occurring phosphatidyl inositol phosphates by sequestering inactive AKT in the cytoplasm and preventing its translocation to the membrane. Therefore it is likely, that the efficiency of these analogs depends on the amount of endogenous PI(4,5)P_2 _and PI(3,4,5)P_3. _Under normal cell culture conditions a broad range of growth factors stimulate signaling pathways, resulting in an increase of PI(3,4,5)P_3 _[26]. Our experiments suggest that the applied concentrations of SH-5 and SH-6 are not sufficient to inhibit the phosphorylation of AKT efficiently in three colorectal cancer cell lines in this context. However, since both compounds have strong structural similarities to PI(4,5)P_2_, they may interact with targets distinct from AKT, e.g. PLC. PLC isoforms are localized to the cleavage furrow and may be involved in the control of the progression through cytokinesis by regulating local PI(4,5)P_2 _levels [17]. Based on the different cellular effects of the specific PLC inhibitor U73122, we conclude that the PIA-induced binucleation is independent on global PLC activity. Nevertheless we cannot exclude the possibility that SH-5 and SH-6 alter the sub cellular localization of PLC during cytokinesis, resulting in a disorganization of the PI(4,5)P_2 _dependent signaling.

Gene expression signatures derived from PIA-treated SW480 cells have a high similarity to those observed in MCF7 cells treated with PKC signaling pathway inhibitors. The PKC protein family consists of at least 10 serine/threonine protein kinases which are involved in the control of a wide variety of cellular processes. Activation of PKCs is mediated by diacylglycerol (DAG), Ca^2+ ^and PDK1, which are influenced by the PI(4,5)P_2 _levels [27]. It was shown that resveratrol inhibits the polyphospho-inositide metabolism in activated platelets resulting in a decrease of the PI(4,5)P_2 _level [28]. We therefore suppose that a similar mechanism contributes to the perturbation of PI(4,5)P_2 _levels in SW480 cells, followed by a decreased PKC activity. Rottlerin is a known inhibitor of PKC δ, pointing at a special role of this isoform during cytokinesis in SW480 cells. Interestingly, we recognized a more than two-fold mRNA expression of PKC δ in SW480 cells as compared to the other cell lines. We can speculate that this expression difference may be partially responsible for the different sensitivity of the cell lines to the treatment with the PIAs.

In this context it is also interesting that the response of SW480 cells to long term LY294002 treatment is different compared to the two other cell lines both at the transcriptional and phenotypic level. Whereas the phosphorylation of AKT was strongly inhibited in 2 hours, it was re-phosphorylated within 48 hours. Experiments with conditioned culture medium exclude the possibility that LY294002 decayed during this time. Even after 48 hours the remaining LY294002 in the culture medium was sufficient to block AKT phosphorylation in prior untreated SW480 cells within two hours (data not shown). It is also remarkable that we detected more transcriptional alterations in the SW480 cells as in the two other cell lines. In contrast to SW480 cells, HT29 and the HCT116 harbor an oncogenic mutation in the PIK3CA gene resulting in an increased PI3 kinase activity. This may compensate for the effects caused by SH-5 and SH-6 [29].

## Conclusions

Due to its multiple functions and oncogenic potential AKT is a promising target for pharmacologic intervention in cancer therapy. The design of phosphoinositide analogues represents a targeted approach towards this issue. Our study characterizes the actions of two phosphoinoistide analogues (SH-5 and SH-6) in human colorectal cancer cell lines. Independent of AKT inhibition SH-5 and SH-6 interfered with important cellular functions contributing to the outcome of the treatment.

## Methods

### Cell lines and cell culture

SW480, HT29 and HCT116 cells were cultured in complete L-15 medium at 37°C and 5% CO_2 _in a humified incubator. Following chemical compounds were used for treament: LY-294002 (20 μM; Alexis Deutschland GmbH, Grünberg, Germany), Wortmannin (1 μM; Sigma) , SH-5 (10 μM; Alexis Deutschland GmbH, Grünberg, Germany), SH-6 (10 μM; Alexis Deutschland GmbH, Grünberg, Germany), U73122 (1, 3 and 10 μM; Merck KGaA, Darmstadt, Germany), Rottlerin and Resveratrol (10 μM and 50 μM, respectively; (Calbiochem) Merck KGaA, Darmstadt, Germany). DMSO served as a negative control unless otherwise specified. The DMSO content of the different experiments was adjusted to a final concentration of 0,29%. Cells were treated for 2 hours, 48 hours or 72 hours.

### Immunoblots

Cells were lysed at the corresponding time points using SDS-lysis buffer (1% SDS, 10 mM Tris-HCl pH 7.5, 2 mM EDTA pH 8). 10 μg of protein of whole cell lysates per lane were fractionated by SDS-PAGE and blotted onto nitrocellulose membranes (Schleicher & Schuell Bioscience GmbH, Dassel, Germany). Following primary antibodies were used: AKT (polyclonal, Cell Signaling Technology Inc., Beverly, USA), Phospho-AKT (phospho-Serine 473; polyclonal; Cell Signaling), and beta-actin (Santa Cruz Biotechnology, Inc., Santa Cruz, USA). For protein detection secondary antibodies coupled to horseradish peroxidase (Cell Signaling Technology Inc., Beverly, USA) and ECL (Amersham Biosciences Europe GmbH, Freiburg, Germany) were applied.

### Cell proliferation

Cells were treated for 24 hrs, 48 hrs and 72 hrs with the inhibitors or DMSO. Cell proliferation was assessed at the corresponding time points using the colorimetric XTT-assay (Roche Diagnostics GmbH, Mannheim, Germany) according to the manufacturer's protocol. The extinction measurements were calculated relative to the negative control at 72 hrs. The means of three independent experiments are presented.

### Fluorescence activated cell sorting (FACS)

Both adherent and floating cells were collected after 48 hrs of treatment and washed twice in phosphate-buffered saline, then fixed overnight using 70% ethanol. Following centrifugation the supernatant was discarded and the cell pellet was resuspended in dilution buffer (0.1% Triton X 100, 0.5% BSA in phosphate buffered saline, supplemented with 80 μg/ml RNase (RNase, DNase free; Roche Diagnostics GmbH, Mannheim, Germany)). Samples were kept at room temperature for 30 min. and then centrifuged. The supernatant was discarded and cells were stained with 20 μg/ml propidium iodide (Fluka, Heidelberg, Germany) in dilution buffer. Samples were analysed by flow cytometry (FACS Calibur System; BD Biosciences, Heidelberg, Germany). Fragments of damaged or apoptotic cells were determined as pre-G1 fraction using WinMDI (WinMDI V. 2.8; Joseph Trotter; freeware). All experiments were performed in triplicate.

### RNA extraction and purification

Following inhibitor-treatment for 48 hours cells were washed twice with ice-cold phosphate buffered saline supplemented with diethylpyrocarbonate and then lysed using Trizol (Invitrogen). The suspension was transferred to a new tube and chloroform was added at a ratio of 1:6. After mixing thoroughly the suspension was centrifuged for 15 min. at 8°C at 12.000 G. The interphase was transferred to fresh tube and an equivalent amount of isopropanol was added. The suspension was inverted several times. Following 10 min. at room temperature samples were centrifuged for 15 min. at 4°C at 12.000 G. The supernatant was discarded, the pellet washed twice with 75% ice-cold ethanol and then dissolved in RNase free water. RNA extracts were further purified using RNeasy-Kit (Qiagen) according to the manufacturer's clean-up protocol.

### Microarray analysis

The human arrays HG-U133A (Affymetrix, Santa Clara, CA, USA) comprised a set of 22,283 known genes. Labelling of RNA targets, hybridization and post-hybridization procedures were performed according to protocols provided by Affymetrix; quality control of RNA extracts was performed using Test-3-Chips (Affymetrix, Santa Clara, CA, USA). Following washing and staining, probe arrays were scanned twice at 3 μm resolution using a confocal scanner with argon laser instrument (Hewlett-Packard, Santa Clara, CA, USA), controlled by Microarray Suite 5.0 software (Affymetrix). Photoemission was detected by a photomultiplier tube through a 570-nm long pass filter. Computer-generated array images were overlaid with a virtual grid controlled by Microarray Suite 5.0 software (Affymetrix, Santa Clara, CA, USA). This step allowed definition of each feature and alignment within known array dimensions. About 40 pixels within each feature were averaged after discarding outliers and pixels near feature boundaries. Gene expression levels were calculated according to the average hybridization intensities of perfectly matched versus mismatched oligonucleotide probes. Arrays were scaled to by Microarray Suite 5.0 software to an average intensity of 2,500 per gene and analyzed independently. Probe sets were either marked absent or present according to their signal intensity and quality of hybridisation. Probe sets which were marked absent in all array experiments were excluded from further analysis. Probe sets which showed at least two fold change in intensity compared to DMSO control were considered up regulated or down regulated respectively.

Microarray data are available at the GEO database http://www.ncbi.nlm.nih.gov/ under the accession number GSE18005.

### RT-PCR

Transcript sequences were obtained from NCBI Entrez Nucleotide http://www.ncbi.nlm.nih.gov/entrez. Primers were designed using primer-3-input http://frodo.wi.mit.edu/primer3/. Primer sequences were checked for unspecific overlap with other transcripts using NCBI Blast http://www.ncbi.nlm.nih.gov/BLAST. To identify unwanted amplification of genomic DNA by product size, primers were checked with Ensembl http://www.ensembl.org/index.html to span introns. Selected primers were synthesized by MWG Biotech (Berlin, Germany). Rt-PCR was performed using Access RT-PCR-Kit (Promega) using 4 ηg of purified RNA. Products were fractioned using agarose gel electrophoresis with ethidiumbromide. Products were analysed under UV-light. Primer sequences and reaction conditions are listed below (see Table 1)

**Table 1:** Primer sequences and  reaction conditions

primer	sequence	Ta*	product size
GAPDH L	5' - CGACCACTTTGTCAAGCTCA - 3'	56°C	205 bp
GAPDH R	5'- AGGGGTCTACATGGCAACTG - 3'	56°C	
ASPM L	5' - CAGTGATGTCACAGGGTTGG - 3'	54°C	272 bp
ASPM R	5' - CTCGTGAAAAAGCCAAAAGG - 3'	54°C	
CENPF L	5' - GCCCATATATCCTGCGAAGA - 3'	56°C	258 bp
CENPF R	5' - GGCTGTCAGTCGGACTTCTC - 3'	56°C	
NUSAP1 L	5' - TGTGCTTGGGACACACAAAT - 3'	54°C	297 bp
NUSAP1 R	5' - CGTTTCTTCCGTTGCTCTTC - 3'	54°C	
PRC1 L	5' - ACAAAGGCTTCTAGGCGTGA - 3'	56°C	290 bp
PRC1 R	5' - GTGGCCACAGCTTCTCTTTC - 3'	56°C	

*annealing temperature

### Fluorescence microscopy

Cells were seeded on cover slides and treated with the inhibitors for 48 hrs. Cells were then washed twice with ice-cold phosphate buffered saline and fixed with pre chilled acetone/methanol (1:1) at -20°C. The cells were pre incubated in PBS containing 1.5% horse serum to block non-specific binding of antibodies. The same buffer was used for all incubation steps. We used the following antibodies for staining of the cells: anti-lamin A/C, anti-vimentin, anti-PRC1 and anti-γ-Tubulin (all: Santa Cruz Biotechnology, Inc., Santa Cruz, USA). In order to detect the DNA we included DAPI (Sigma, Germany) in the last incubation step. Bound antibodies and stained DNA were detected using a confocal laser scanning microscope from Leica (DM IRBE). For quantification of binucleation, 200-300 nuclei per sample were counted. Three independent experiments were performed, each counted by at least two independent, blinded investigators and the means are presented.

### Time lapse recording

We used the Biozero microscope from Keyence (Neu-Isenburg, Germany) equipped with a time lapse unit. We started 24 hours after adding the PIAs or DMSO to take pictures every 30 seconds. Pictures were aligned to a movie with a frequency of 25 pictures per second using the free software JPGVideo (NDW Ltd., Nottingham, England). Cutting and cropping of the movies were done with the free software VirtualDub 1.8.8.

### Statistical analysis

Statistical analysis of the number of binucleated cells was performed using Students t-Test. A p-value < 0.05 was considered significant. For the GO analysis, we used the implemented statistical features of Expander 4.0 with an adjusted p-value < 0.05.

## Competing interests

The authors declare that they have no competing interests.

## Authors' contributions

TK designed and performed experiments, analyzed data and wrote the paper; MT and EH performed experiments; RS conceived the study and wrote the paper, KJ conceived the study, designed and performed experiments, analyzed data, supervised the project and wrote the paper. All authors read and approved the final manuscript.

## Pre-publication history

The pre-publication history for this paper can be accessed here:

http://www.biomedcentral.com/1471-2407/10/287/prepub

## Supplementary Material

Additional file 1 **Overrepresented GO-annotations**. We subjected sets of up- or down-regulated genes to an analysis of functional overrepresentation using Expander 4.0. The Excel file shows the summary of overrepresented GO - annotations, considering a corrected p-value < 0.05 as significant. Click here for file

Additional file 2 **Cell growth and apoptosis**. (A, C, E) The cell growth of the three colorectal cancer cell lines was determined 0, 24, 48, and 72 hours of incubation with the indicated inhibitors using a colorimetric XTT assay. (B, D, F) Cells were incubated for 48 hours with either one of the inhibitors or DMSO as a control. The cells were labeled with propidium iodide after fixation. Events in front of the G1 peak of the histograms were gated and displayed as percent in the graphs. Click here for file

Additional file 3 **Time lapse recording of control SW480 cells**. The time lapse movies correspond to Figure 6A. Pictures were taken every 30 seconds and aligned to a movie with 25 pictures per second. The movie demonstrates the normal cell division of control SW480 cells. Click here for file

Additional file 4 **Time lapse recording of SH-6 treated SW480 cells**. The time lapse movies correspond to Figure 6B. Pictures were taken every 30 seconds and aligned to a movie with 25 pictures per second. The movie shows abscission defects in SW480 cells treated with SH-6. Click here for file

Additional file 5 **Time lapse recording of SH-6 treated SW480 cells**. The time lapse movies correspond to Figure 6C. Pictures were taken every 30 seconds and aligned to a movie with 25 pictures per second. The movie shows abscission defects in SW480 cells treated with SH-6. Click here for file

Additional file 6 **Time lapse recording of SH-6 treated SW480 cells**. The time lapse movies correspond to Figure 6D. Pictures were taken every 30 seconds and aligned to a movie with 25 pictures per second. The movie shows abscission defects in SW480 cells treated with SH-6. Click here for file

Additional file 7 **Summary of Connectivity Map analysis (SH-5)**. Signatures of up- and down-regulated genes of SW480 cells preincubated with SH-5 were compared with a collection of gene expression profiles derived from treatment of different cell lines with more than 1300 compounds, resulting in 6100 individual treatment instances (Connectivity Map (build 02)). The supplementary tables provide the permutated results for the 50 highest ranking substances. Click here for file

Additional file 8 **Summary of Connectivity Map analysis (SH-6)**. Signatures of up- and down-regulated genes of SW480 cells preincubated with SH-6 were compared with a collection of gene expression profiles derived from treatment of different cell lines with more than 1300 compounds, resulting in 6100 individual treatment instances (Connectivity Map (build 02)). The supplementary tables provide the permutated results for the 50 highest ranking substances. Click here for file
