# Supplementary material for: Characterization of AKT independent effects of the synthetic AKT inhibitors SH-5 and SH-6 using an integrated approach combining transcriptomic profiling and signaling pathway perturbations
Source: BMC Cancer. 2010 Jun 14;10:287. doi: 10.1186/1471-2407-10-287 (PMC2895615; doi:10.1186/1471-2407-10-287)
Supplement: Additional file 1 — Overrepresented GO-annotations. We subjected sets of up- or down-regulated genes to an analysis of functional overrepresentation using Expander 4.0. The Excel file shows the summary of overrepresented GO - annotations, considering a corrected p-value < 0.05 as significant. [file 1471-2407-10-287-S1.DOC]

| **Cell line** | **Set** | **GO-term** | **Description** | **Raw p-value** | **Corrected p-value** | **Coverage** | **Frequency** |
| --- | --- | --- | --- | --- | --- | --- | --- |
| SW480 | Down SH-5 and SH-6 | GO:0007049 | cell cycle | 3.06E-16 | 0.001 | 46 | 0.20627803 |
|  |  | GO:0051301 | cell division | 7.02E-15 | 0.001 | 23 | 0.10313901 |
|  |  | GO:0000278 | mitotic cell cycle | 5.41E-14 | 0.001 | 25 | 0.11210762 |
|  |  | GO:0000279 | M phase | 1.37E-12 | 0.001 | 22 | 0.09865471 |
|  |  | GO:0044430 | cytoskeletal part | 2.03E-08 | 0.001 | 25 | 0.11210762 |
|  |  | GO:0007051 | spindle organization and biogenesis | 2.95E-08 | 0.001 | 7 | 0.03139013 |
|  |  | GO:0007017 | microtubule-based process | 8.99E-08 | 0.001 | 14 | 0.06278027 |
|  |  | GO:0007010 | cytoskeleton organization and biogenesis | 1.75E-07 | 0.001 | 22 | 0.09865471 |
|  |  | GO:0016043 | cellular component organization and biogenesis | 3.30E-07 | 0.001 | 61 | 0.2735426 |
|  |  | GO:0043232 | intracellular non-membrane-bound organelle | 4.08E-07 | 0.001 | 43 | 0.19282511 |
|  |  | GO:0050794 | regulation of cellular process | 1.07E-06 | 0.002 | 80 | 0.35874438 |
|  |  | GO:0043687 | post-translational protein modification | 1.22E-05 | 0.006 | 36 | 0.16143498 |
|  |  | GO:0044446 | intracellular organelle part | 2.06E-05 | 0.011 | 53 | 0.23766816 |
|  |  |  |  |  |  |  |  |
| SW480 | Down SH-5 | GO:0051301 | cell division | 1.05E-13 | 0.001 | 26 | 0.07624634 |
|  |  | GO:0007049 | cell cycle | 2.11E-13 | 0.001 | 53 | 0.15542522 |
|  |  | GO:0000278 | mitotic cell cycle | 2.53E-12 | 0.001 | 28 | 0.08211144 |
|  |  | GO:0022403 | cell cycle phase | 4.41E-12 | 0.001 | 29 | 0.08504399 |
|  |  | GO:0007051 | spindle organization and biogenesis | 4.77E-07 | 0.002 | 7 | 0.02052786 |
|  |  | GO:0043232 | intracellular non-membrane-bound organelle | 2.52E-06 | 0.007 | 55 | 0.16129032 |
|  |  | GO:0050794 | regulation of cellular process | 4.03E-06 | 0.010 | 109 | 0.3196481 |
|  |  | GO:0016043 | cellular component organization and biogenesis | 4.87E-06 | 0.010 | 79 | 0.23167156 |
|  |  | GO:0007017 | microtubule-based process | 1.08E-05 | 0.015 | 14 | 0.04105572 |
|  |  | GO:0048523 | negative regulation of cellular process | 1.22E-05 | 0.015 | 42 | 0.12316716 |
|  |  | GO:0044430 | cytoskeletal part | 3.19E-05 | 0.038 | 25 | 0.07331378 |
|  |  | GO:0005634 | nucleus | 3.59E-05 | 0.039 | 109 | 0.3196481 |
|  |  |  |  |  |  |  |  |
| SW480 | Down SH-6 | GO:0007049 | cell cycle | 1.04E-26 | 0.001 | 115 | 0.15013056 |
|  |  | GO:0000278 | mitotic cell cycle | 8.42E-25 | 0.001 | 60 | 0.07832898 |
|  |  | GO:0000279 | M phase | 3.84E-24 | 0.001 | 55 | 0.07180157 |
|  |  | GO:0022403 | cell cycle phase | 4.62E-24 | 0.001 | 62 | 0.08093995 |
|  |  | GO:0000087 | M phase of mitotic cell cycle | 5.09E-24 | 0.001 | 49 | 0.06396867 |
|  |  | GO:0005634 | nucleus | 1.97E-18 | 0.001 | 280 | 0.36553526 |
|  |  | GO:0051301 | cell division | 1.63E-16 | 0.001 | 42 | 0.05483029 |
|  |  | GO:0043232 | intracellular non-membrane-bound organelle | 3.16E-15 | 0.001 | 132 | 0.17232376 |
|  |  | GO:0005694 | chromosome | 9.34E-14 | 0.001 | 48 | 0.06266318 |
|  |  | GO:0006259 | DNA metabolic process | 1.02E-12 | 0.001 | 76 | 0.09921671 |
|  |  | GO:0044422 | organelle part | 4.26E-12 | 0.001 | 172 | 0.22454308 |
|  |  | GO:0051276 | chromosome organization and biogenesis | 4.83E-12 | 0.001 | 49 | 0.06396867 |
|  |  | GO:0016043 | cellular component organization and biogenesis | 1.44E-11 | 0.001 | 178 | 0.23237598 |
|  |  | GO:0006996 | organelle organization and biogenesis | 3.29E-11 | 0.001 | 94 | 0.12271541 |
|  |  | GO:0000070 | mitotic sister chromatid segregation | 3.33E-10 | 0.001 | 12 | 0.0156658 |
|  |  | GO:0050794 | regulation of cellular process | 4.58E-09 | 0.001 | 234 | 0.30548304 |
|  |  | GO:0044430 | cytoskeletal part | 1.29E-08 | 0.001 | 53 | 0.0691906 |
|  |  | GO:0015630 | microtubule cytoskeleton | 1.70E-08 | 0.001 | 38 | 0.04960835 |
|  |  | GO:0000166 | nucleotide binding | 1.93E-08 | 0.001 | 137 | 0.17885117 |
|  |  | GO:0003677 | DNA binding | 3.65E-08 | 0.001 | 135 | 0.1762402 |
|  |  | GO:0007051 | spindle organization and biogenesis | 5.66E-08 | 0.001 | 10 | 0.01305483 |
|  |  | GO:0005524 | ATP binding | 8.91E-08 | 0.001 | 99 | 0.12924282 |
|  |  | GO:0006974 | response to DNA damage stimulus | 2.20E-07 | 0.001 | 35 | 0.04569191 |
|  |  | GO:0000075 | cell cycle checkpoint | 6.82E-07 | 0.001 | 13 | 0.01697128 |
|  |  | GO:0044428 | nuclear part | 1.23E-06 | 0.001 | 71 | 0.0926893 |
|  |  | GO:0043687 | post-translational protein modification | 2.58E-06 | 0.002 | 93 | 0.12140992 |
|  |  | GO:0007017 | microtubule-based process | 1.11E-05 | 0.007 | 22 | 0.02872063 |
|  |  | GO:0006310 | DNA recombination | 1.12E-05 | 0.007 | 16 | 0.02088773 |
|  |  | GO:0003682 | chromatin binding | 2.39E-05 | 0.021 | 15 | 0.01958225 |
|  |  |  |  |  |  |  |  |
| SW480 | Up SH-5 and SH-6 | GO:0016875 | ligase activity\, forming carbon-oxygen bonds | 1.88E-06 | 0.002 | 7 | 0.03517588 |
|  |  | GO:0005625 | soluble fraction | 2.33E-06 | 0.002 | 14 | 0.07035176 |
|  |  | GO:0005507 | copper ion binding | 1.03E-05 | 0.007 | 7 | 0.03517588 |
|  |  | GO:0016477 | cell migration | 1.40E-05 | 0.009 | 12 | 0.06030151 |
|  |  | GO:0030334 | regulation of cell migration | 2.23E-05 | 0.012 | 6 | 0.03015075 |
|  |  | GO:0009308 | amine metabolic process | 2.26E-05 | 0.012 | 16 | 0.08040201 |
|  |  |  |  |  |  |  |  |
| SW480 | Up SH-5 | GO:0016875 | ligase activity\, forming carbon-oxygen bonds | 1.92E-07 | 0.001 | 9 | 0.03010033 |
|  |  | GO:0005625 | soluble fraction | 3.36E-06 | 0.003 | 17 | 0.05685619 |
|  |  | GO:0000323 | lytic vacuole | 5.08E-06 | 0.003 | 13 | 0.04347826 |
|  |  | GO:0009308 | amine metabolic process | 9.65E-06 | 0.009 | 21 | 0.07023411 |
|  |  | GO:0006519 | amino acid and derivative metabolic process | 1.92E-05 | 0.014 | 18 | 0.06020067 |
|  |  | GO:0000049 | tRNA binding | 3.25E-05 | 0.028 | 4 | 0.01337793 |
|  |  |  |  |  |  |  |  |
| SW480 | Up SH-6 | GO:0006412 | translation | 1.36E-07 | 0.001 | 30 | 0.06912442 |
|  |  | GO:0016875 | ligase activity\, forming carbon-oxygen bonds | 3.63E-07 | 0.002 | 10 | 0.02304148 |
|  |  | GO:0000323 | lytic vacuole | 5.63E-07 | 0.002 | 17 | 0.03917051 |
|  |  | GO:0005625 | soluble fraction | 6.21E-07 | 0.002 | 22 | 0.05069124 |
|  |  | GO:0009308 | amine metabolic process | 3.81E-06 | 0.004 | 27 | 0.06221198 |
|  |  | GO:0044444 | cytoplasmic part | 6.17E-06 | 0.006 | 105 | 0.24193548 |
|  |  | GO:0044445 | cytosolic part | 6.62E-06 | 0.006 | 13 | 0.02995392 |
|  |  | GO:0015934 | large ribosomal subunit | 1.66E-05 | 0.012 | 8 | 0.01843318 |
|  |  | GO:0005507 | copper ion binding | 2.94E-05 | 0.025 | 9 | 0.02073733 |
|  |  | GO:0019752 | carboxylic acid metabolic process | 3.34E-05 | 0.028 | 29 | 0.06682028 |
|  |  |  |  |  |  |  |  |
|  |  |  |  |  |  |  |  |
| HT29 | Up SH-5 and SH-6 | GO:0006955 | immune response | 2.41E-12 | 0.001 | 28 | 0.18064517 |
|  |  | GO:0009615 | response to virus | 1.26E-10 | 0.001 | 12 | 0.07741936 |
|  |  |  |  |  |  |  |  |
| HT29 | Up SH-5 | GO:0006955 | immune response | 2.30E-10 | 0.001 | 30 | 0.14084508 |
|  |  | GO:0009615 | response to virus | 4.64E-09 | 0.001 | 12 | 0.05633803 |
|  |  |  |  |  |  |  |  |
| HT29 | Up SH-6 | GO:0006955 | immune response | 3.07E-12 | 0.001 | 37 | 0.13962264 |
|  |  | GO:0009615 | response to virus | 4.70E-11 | 0.001 | 15 | 0.05660377 |
|  |  | GO:0042127 | regulation of cell proliferation | 4.53E-05 | 0.036 | 20 | 0.0754717 |
|  |  |  |  |  |  |  |  |
|  |  |  |  |  |  |  |  |
| HCT116 | Down SH-6 | GO:0006996 | organelle organization and biogenesis | 5.06E-06 | 0.003 | 61 | 0.10931899 |
|  |  | GO:0016043 | cellular component organization and biogenesis | 1.64E-05 | 0.015 | 116 | 0.20788531 |
|  |  |  |  |  |  |  |  |
| HCT116 | Up SH-5 and SH-6 | GO:0008203 | cholesterol metabolic process | 4.50E-05 | 0.012 | 4 | 0.10810811 |
|  |  |  |  |  |  |  |  |
| HCT116 | Up SH-5 | GO:0016126 | sterol biosynthetic process | 3.07E-09 | 0.001 | 7 | 0.0673077 |
|  |  | GO:0016125 | sterol metabolic process | 6.03E-09 | 0.001 | 9 | 0.08653846 |
|  |  | GO:0006720 | isoprenoid metabolic process | 1.11E-05 | 0.004 | 4 | 0.03846154 |

| HCT116 | Down Ly294002 | GO:0006520 | amino acid metabolic process | 9.24E-09 | 0.001 | 13 | 0.12264151 |
| --- | --- | --- | --- | --- | --- | --- | --- |
|  |  | GO:0016875 | ligase activity\, forming carbon-oxygen bonds | 6.84E-07 | 0.001 | 6 | 0.05660377 |
|  |  | GO:0005386 | carrier activity | 2.86E-05 | 0.011 | 10 | 0.09433962 |
|  |  | GO:0009058 | biosynthetic process | 4.57E-05 | 0.016 | 20 | 0.18867925 |
|  |  | GO:0000267 | cell fraction | 7.62E-05 | 0.029 | 17 | 0.16037735 |
|  |  |  |  |  |  |  |  |
| HCT116 | Up Wortmanin | GO:0006468 | protein amino acid phosphorylation | 6.06E-05 | 0.019 | 12 | 0.12371134 |
|  |  |  |  |  |  |  |  |
| HT29 | Up Ly294002 | GO:0004672 | protein kinase activity | 2.95E-05 | 0.025 | 27 | 0.07964602 |
|  |  |  |  |  |  |  |  |
| SW480 | Down Ly294002 | GO:0008092 | cytoskeletal protein binding | 2.02E-05 | 0.014 | 27 | 0.05793991 |
