# Supplementary figures and images for: Characterization of AKT independent effects of the synthetic AKT inhibitors SH-5 and SH-6 using an integrated approach combining transcriptomic profiling and signaling pathway perturbations
Source: BMC Cancer. 2010 Jun 14;10:287. doi: 10.1186/1471-2407-10-287 (PMC2895615; doi:10.1186/1471-2407-10-287)

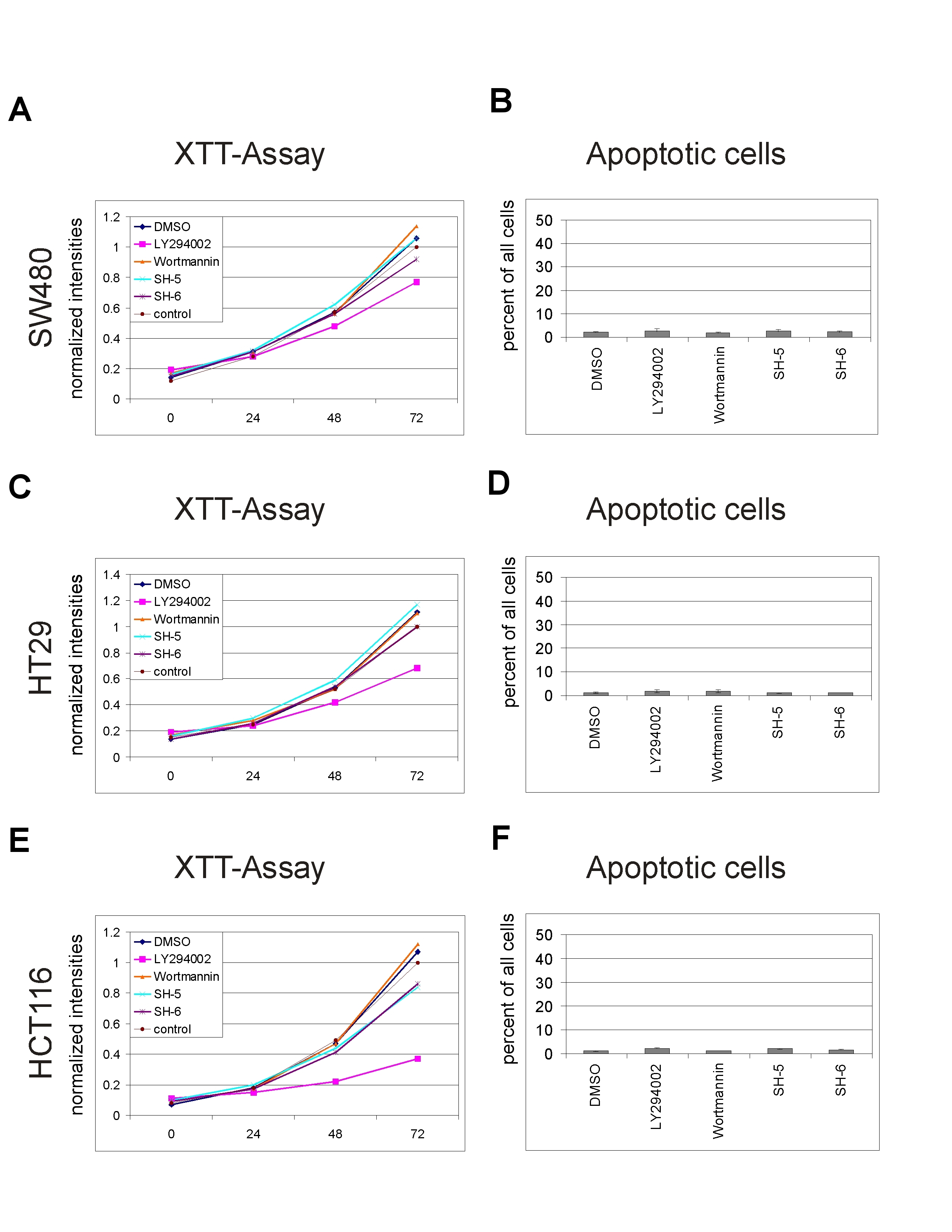

Supplement: Additional file 2 — Cell growth and apoptosis. (A, C, E) The cell growth of the three colorectal cancer cell lines was determined 0, 24, 48, and 72 hours of incubation with the indicated inhibitors using a colorimetric XTT assay. (B, D, F) Cells were incubated for 48 hours with either one of the inhibitors or DMSO as a control. The cells were labeled with propidium iodide after fixation. Events in front of the G1 peak of the histograms were gated and displayed as percent in the graphs. [file 1471-2407-10-287-S2.JPEG]
