# Supplementary material for: Characterization of AKT independent effects of the synthetic AKT inhibitors SH-5 and SH-6 using an integrated approach combining transcriptomic profiling and signaling pathway perturbations
Source: BMC Cancer. 2010 Jun 14;10:287. doi: 10.1186/1471-2407-10-287 (PMC2895615; doi:10.1186/1471-2407-10-287)
Supplement: Additional file 7 — Summary of Connectivity Map analysis (SH-5). Signatures of up- and down-regulated genes of SW480 cells preincubated with SH-5 were compared with a collection of gene expression profiles derived from treatment of different cell lines with more than 1300 compounds, resulting in 6100 individual treatment instances (Connectivity Map (build 02)). The supplementary tables provide the permutated results for the 50 highest ranking substances. [file 1471-2407-10-287-S7.DOC]

| rank | cmap name | mean | n | enrichment | p | specificity | percent non-null |
| --- | --- | --- | --- | --- | --- | --- | --- |
| 1 | valinomycin | 0.652 | 4 | 0.961 | 0 | 0.0116 | 100 |
| 2 | 5707885 | 0.738 | 4 | 0.96 | 0 | 0 | 100 |
| 3 | erastin | 0.62 | 4 | 0.947 | 0 | 0 | 100 |
| 4 | phenoxybenzamine | 0.709 | 4 | 0.94 | 0 | 0.1386 | 100 |
| 5 | gossypol | 0.63 | 6 | 0.921 | 0 | 0 | 100 |
| 6 | prochlorperazine | 0.473 | 16 | 0.624 | 0 | 0.0437 | 87 |
| 7 | thioridazine | 0.461 | 20 | 0.575 | 0 | 0.1553 | 75 |
| 8 | trifluoperazine | 0.44 | 16 | 0.59 | 0.00002 | 0.1154 | 68 |
| 9 | thapsigargin | 0.684 | 3 | 0.97 | 0.00004 | 0.0764 | 100 |
| 10 | semustine | 0.578 | 4 | 0.923 | 0.00004 | 0.017 | 100 |
| 11 | econazole | 0.625 | 4 | 0.919 | 0.00004 | 0.0103 | 100 |
| 12 | **resveratrol** | 0.471 | 9 | 0.7 | 0.00006 | 0.0931 | 88 |
| 13 | **rottlerin** | 0.687 | 3 | 0.954 | 0.0001 | 0.0259 | 100 |
| 14 | niclosamide | 0.664 | 5 | 0.86 | 0.0001 | 0.0105 | 100 |
| 15 | mestranol | 0.508 | 4 | 0.888 | 0.00016 | 0 | 100 |
| 16 | clioquinol | 0.567 | 5 | 0.847 | 0.00018 | 0.0157 | 100 |
| 17 | puromycin | 0.528 | 4 | 0.88 | 0.0003 | 0.0843 | 100 |
| 18 | lomustine | 0.496 | 4 | 0.874 | 0.00034 | 0.0471 | 100 |
| 19 | cloperastine | 0.493 | 6 | 0.769 | 0.00042 | 0.0052 | 83 |
| 20 | dipyridamole | 0.498 | 6 | 0.751 | 0.00058 | 0 | 100 |
| 21 | azacitidine | 0.592 | 3 | 0.929 | 0.00066 | 0.0529 | 100 |
| 22 | ionomycin | 0.565 | 3 | 0.927 | 0.00072 | 0.0105 | 100 |
| 23 | 5224221 | 0.691 | 2 | 0.978 | 0.00082 | 0.0894 | 100 |
| 24 | oxamniquine | -0.473 | 4 | -0.854 | 0.00082 | 0 | 75 |
| 25 | astemizole | 0.664 | 5 | 0.788 | 0.00092 | 0.0758 | 80 |
| 26 | primaquine | 0.43 | 4 | 0.841 | 0.00101 | 0 | 100 |
| 27 | fluphenazine | 0.349 | 18 | 0.435 | 0.00115 | 0.1865 | 61 |
| 28 | equilin | 0.482 | 5 | 0.772 | 0.00136 | 0 | 100 |
| 29 | homosalate | -0.378 | 4 | -0.833 | 0.00145 | 0 | 50 |
| 30 | meglumine | -0.262 | 4 | -0.832 | 0.00145 | 0 | 50 |
| 31 | butoconazole | 0.526 | 4 | 0.822 | 0.00167 | 0 | 100 |
| 32 | enilconazole | 0.471 | 4 | 0.822 | 0.00173 | 0 | 100 |
| 33 | harmol | -0.565 | 4 | -0.822 | 0.00189 | 0.0273 | 75 |
| 34 | clotrimazole | 0.59 | 5 | 0.756 | 0.00202 | 0.0278 | 100 |
| 35 | monensin | 0.331 | 6 | 0.693 | 0.00213 | 0.0794 | 66 |
| 36 | cisapride | 0.453 | 4 | 0.814 | 0.00231 | 0 | 100 |
| 37 | felodipine | 0.45 | 7 | 0.639 | 0.00236 | 0.0099 | 85 |
| 38 | perphenazine | 0.583 | 5 | 0.746 | 0.0024 | 0.0516 | 100 |
| 39 | pyrvinium | 0.584 | 6 | 0.687 | 0.00242 | 0.1395 | 83 |
| 40 | propofol | 0.507 | 4 | 0.806 | 0.00265 | 0 | 100 |
| 41 | quinisocaine | 0.545 | 4 | 0.803 | 0.00284 | 0.0054 | 100 |
| 42 | AG-013608 | 0.264 | 8 | 0.595 | 0.00293 | 0.0142 | 62 |
| 43 | kaempferol | 0.423 | 4 | 0.798 | 0.00318 | 0.0052 | 100 |
| 44 | nicergoline | 0.485 | 5 | 0.723 | 0.00374 | 0.0111 | 100 |
| 45 | monastrol | 0.266 | 8 | 0.582 | 0.00399 | 0.0125 | 87 |
| 46 | 5253409 | 0.573 | 2 | 0.952 | 0.00419 | 0.0103 | 100 |
| 47 | miconazole | 0.488 | 5 | 0.711 | 0.00471 | 0 | 100 |
| 48 | chlorcyclizine | 0.437 | 6 | 0.657 | 0.00491 | 0.025 | 83 |
| 49 | methylbenzethonium chloride | 0.412 | 6 | 0.654 | 0.00514 | 0.0524 | 66 |
| 50 | triamterene | 0.343 | 5 | 0.705 | 0.00537 | 0.0134 | 80 |
